# Interstitial Nephritis Induced by Repeated Nonsteroidal Anti-inflammatory Drugs (NSAIDs) Use for Persistent Fever: A Case Report

**DOI:** 10.7759/cureus.87304

**Published:** 2025-07-04

**Authors:** Norihito Yoshida, Yusuke Suzuki, Mai Hitaka, Keisuke Yamazaki, Yasushi Ohashi

**Affiliations:** 1 Nephrology, Toho University Sakura Medical Center, Sakura, JPN

**Keywords:** acute interstitial nephritis (ain), #aki, drug induced aki, n-acetyl glucosaminidase(nag), nsaid abuse, steroid use

## Abstract

Nonsteroidal anti-inflammatory drugs (NSAIDs) are extensively utilized for their analgesic and anti-inflammatory efficacy, yet they pose a significant risk for renal adverse events, notably drug-induced acute interstitial nephritis (DI-AIN). Prompt recognition and appropriate management are paramount to prevent irreversible kidney damage. We present the case of a 46-year-old male with NSAID-induced DI-AIN, emphasizing the diagnostic utility of a specific urinary biomarker profile and the rationale for empirical steroid therapy initiated before histopathological confirmation.

Our patient developed acute kidney injury (AKI) following daily ibuprofen administration for persistent fever. Despite ibuprofen discontinuation on day eight, renal function failed to improve, necessitating hospital admission on day 14. On day 15, his serum creatinine (Cr) level was 1.86 mg/dL. Urinalysis revealed mild proteinuria [urine protein-to-creatinine ratio (UPCR): 0.24 g/gCr] but strikingly elevated urinary tubular injury markers: N-acetyl-β-D-glucosaminidase (NAG): 20.9 U/L (on day one), β2-microglobulin (β2MG): 6028 μg/L, and L-type fatty acid-binding protein (L-FABP): 27.75 ng/mL. Based on a strong clinical suspicion of DI-AIN, a kidney biopsy was performed on day 15, and oral prednisolone (PSL, 0.8 mg/kg/day) was commenced the same evening before biopsy results were available. Serum creatinine improved to 1.56 mg/dL by discharge on day 23. Post-discharge, kidney biopsy results confirmed AIN. PSL was gradually tapered and discontinued after approximately 10 months, with sustained renal function recovery (serum creatinine: ~1.1 mg/dL).

This report underscores the importance of suspecting DI-AIN in patients with AKI and a history of NSAID exposure. The pronounced elevation of urinary tubular markers, despite only mild proteinuria, was pivotal in raising clinical suspicion. The negative autoimmune serology further strengthened the diagnosis of a drug-induced etiology. Empirical steroid therapy, initiated due to compelling clinical evidence before histopathological confirmation, appeared to be an effective intervention. While this single case cannot establish a therapeutic standard, it illustrates a clinical scenario where early, empirically-guided treatment may be justified. Kidney biopsy remains indispensable for definitive diagnosis. The report also highlights the pressing need for enhanced patient education on appropriate NSAID utilization. Repeated use of common NSAIDs can precipitate DI-AIN. A diagnostic profile of elevated urinary tubular markers with only mild proteinuria can be a key indicator for suspecting this condition. Empirical steroid therapy, guided by strong clinical suspicion, can be an effective early intervention, with subsequent kidney biopsy providing definitive diagnostic validation. Enhanced patient education on appropriate NSAID use is essential.

## Introduction

Nonsteroidal anti-inflammatory drugs (NSAIDs) represent a cornerstone of therapy for numerous pain and inflammatory conditions, thanks to their potent analgesic and anti-inflammatory properties. Their clinical utility has led to widespread global use across a broad patient demographic, driven by both physician prescriptions and over-the-counter availability, leading to their extensive use in daily life and clinical practice [1-3]. In Japan, this ubiquity is mirrored in reports indicating that the majority of patients with chronic pain conditions, such as osteoarthritis and low back pain, receive NSAID therapy. However, this extensive use is associated with a significant risk of severe renal dysfunction, most notably drug-induced acute interstitial nephritis (DI-AIN). DI-AIN, a condition characterized by inflammatory cellular infiltration of the renal interstitium, is a well-established complication of NSAID therapy. Therefore, cautious administration and vigilant monitoring are imperative, particularly in high-risk populations such as the elderly, patients with pre-existing chronic kidney disease (CKD), heart failure, or those receiving concomitant diuretic therapy, in whom NSAID-induced renal injury occurs with greater frequency and severity.

We present the case of a 46-year-old male with acute kidney injury (AKI) following daily NSAID administration, where a strong clinical suspicion of DI-AIN was raised by a characteristic profile of markedly elevated urinary tubular injury markers in the setting of only mild proteinuria. Steroid therapy was initiated empirically before the diagnosis was subsequently confirmed by kidney biopsy. This report aims to highlight the diagnostic clues and management strategy for NSAID-induced renal injury, emphasizing the importance of appropriate NSAID stewardship and early recognition of DI-AIN.

## Case presentation

A 46-year-old male with a past medical history significant only for migraines presented to our hospital. His illness had begun one month before presentation, when he had developed upper respiratory symptoms after inhaling a large amount of dust while cleaning his house. For these persistent symptoms, he had begun taking ibuprofen three times daily, a medication he typically used only on an as-needed basis. As his symptoms had failed to improve, he had been referred to the Department of Respiratory Medicine on day one. Initial laboratory investigations at this first visit had been significant for acute kidney injury (AKI) and inflammation. Key results included a serum creatinine (Cr) of 1.60 mg/dL, an elevated C-reactive protein (CRP) of 3.09 mg/dL, mild proteinuria [urine protein-to-creatinine ratio (UPCR) 0.24 g/gCr], and markedly elevated urinary tubular injury markers, including N-acetyl-β-D-glucosaminidase (NAG), β2-microglobulin (β2MG), and L-type fatty acid-binding protein (L-FABP) (Table 1).

**Table 1:** Laboratory findings The results indicate acute kidney injury with a serum creatinine of 1.60 mg/dL and an eGFR of 39 mL/min/1.73m². Notable findings include mild leukopenia, elevated inflammatory markers (CRP, γ-GTP, CH50), and hypergammaglobulinemia (IgG). Urinalysis was significant for mild proteinuria (UPCR: 0.24 g/gCr) and markedly elevated levels of urinary tubular injury markers (NAG, β2MG, L-FABP). The autoimmune serology, including ANA and ANCA, was unremarkable. The NAG value is from the initial consultation on day one WBC: white blood cell count; RBC: red blood cell count; Hb: hemoglobin; Ht: hematocrit; Plt: platelets; Alb: Albumin; T-Bil: total bilirubin; AST: aspartate aminotransferase; ALT: alanine aminotransferase; LDH: lactate dehydrogenase; γ-GTP: gamma-glutamyl transferase; BUN: blood urea nitrogen; Cr: creatinine; UA: uric acid; CRP: C-reactive protein; Glu: glucose; HbA1c: hemoglobin A1c (NGSP); Na: sodium; K: potassium; Cl: chloride; Ca: calcium; P: phosphorus; BNP: brain natriuretic peptide; TSH: thyroid-stimulating hormone; FT3: free triiodothyronine; FT4: free thyroxine; IgG: immunoglobulin G; IgA: immunoglobulin A; IgM: immunoglobulin M; C3: complement component 3; C4: complement component 3; CH50: total hemolytic complement; ANA: antinuclear antibody; Anti-dsDNA: anti-double-stranded DNA antibody; MPO-ANCA: myeloperoxidase-antineutrophil cytoplasmic antibody; PR3-ANCA: proteinase 3-antineutrophil cytoplasmic antibody; qual.: qualitative; HPF: high-power field; UPCR: urine protein-to-creatinine ratio; NAG: N-acetyl-β-D-glucosaminidase; β2MG: β2-microglobulin; L-FABP: L-type fatty acid-binding protein

Lab findings		
Blood test item	Patient value	Reference range
WBC	3,390/μL	4,000-10,000/μL
RBC	4.31 million/μL	4.5-5.9 million/μL
Hb	12.9 g/dL	13.5-17.5 g/dL
Ht	38.70%	40-50%
Plt	340,000/μL	150,000-400,000/μL
Alb	4.0 g/dL	4.0-5.0 g/dL
T-Bil	0.6 mg/dL	0.2-1.2 mg/dL
AST	23 IU/L	13-30 IU/L
ALT	27 IU/L	10-40 IU/L
LDH	156 IU/L	120-245 IU/L
γ-GTP	56 IU/L	10-50 IU/L
BUN	14.9 mg/dL	8-20 mg/dL
Cr	1.60 mg/dL	0.6-1.2 mg/dL
UA	4.2 mg/dL	3.0-7.0 mg/dL
CRP	3.09 mg/dL	<0.3 mg/dL
Glu	96 mg/dL	70-109 mg/dL
HbA1c	5.90%	<5.7%
Na	139 mEq/L	135-145 mEq/L
K	3.7 mEq/L	3.5-5.0 mEq/L
Cl	105 mEq/L	98-107 mEq/L
Ca	9.4 mg/dL	8.5-10.5 mg/dL
P	2.9 mg/dL	2.5-4.5 mg/dL
BNP	<5.8 pg/mL	<18.4 pg/mL
TSH	1.75 μIU/mL	0.4-4.0 μIU/mL
FT3	2.62 pg/mL	2.0-4.4 pg/mL
FT4	1.30 ng/dL	0.8-1.8 ng/dL
IgG	1946 mg/dL	700-1600 mg/dL
IgA	248 mg/dL	110-410 mg/dL
IgM	94 mg/dL	40-230 mg/dL
C3	158 mg/dL	90-180 mg/dL
C4	39 mg/dL	10-40 mg/dL
CH50	56.7 U/mL	30-46 U/mL
ANA	<40 (speckled)	<40
Anti-dsDNA	1.8 IU/mL	<30 IU/mL
MPO-ANCA	Negative	Negative
Urinary test item	Patient value	Reference range
Protein (qual.)	1+	Negative
Occult blood	Negative	Negative
Sediment (RBC)	<1/HPF	0-4/HPF
Sediment (WBC)	1-4/HPF	0-4/HPF
UPCR	0.24 g/gCr	<0.15 g/gCr
NAG	20.9 U/L	<11.5 U/L
β2MG	6028 μg/L	<300 μg/L
L-FABP	27.75 ng/mL	<8.4 ng/mL

**Figure 1:**
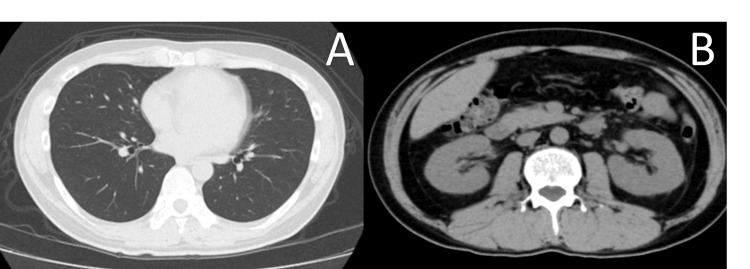
Chest and abdominal CT on admission (A) Non-contrast chest CT. The axial CT image of the chest shows clear lung fields bilaterally, with no evidence of pulmonary infiltrates, consolidation, or pleural effusion. This finding rules out significant acute pulmonary pathology, such as pneumonia, as a cause for the patient's symptoms. (B) Non-contrast abdominal CT focusing on the kidneys. The axial CT image of the abdomen demonstrates the normal size and contour of both kidneys. There is no evidence of hydronephrosis, renal calculi, or significant cortical atrophy, which argues against obstructive uropathy or pre-existing chronic structural kidney disease CT: computed tomography

In light of these results, he was referred to our outpatient nephrology clinic on day eight. Ibuprofen-induced renal injury was highly suspected, and the medication was discontinued. However, as his renal function failed to improve, he was admitted to our hospital for further evaluation and treatment on day 14. On admission, the patient’s physical examination was largely unremarkable. He was alert and oriented with a temperature of 37.0 °C and stable vital signs. Diagnostic imaging, including a chest radiograph and a non-contrast CT scan of the chest and pelvis, revealed no significant abnormalities or alternative etiologies for his condition (Figure 1).

**Figure 2:**
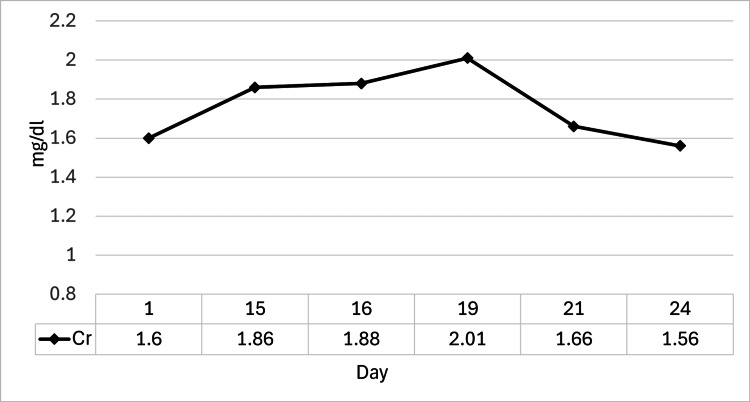
Serum creatinine trend during hospitalization. Following admission, the serum Cr level increased to a peak of 2.01 mg/dL. After the initiation of prednisolone therapy, the Cr level began to decrease, reaching 1.56 mg/dL by the time of discharge Cr: creatinine

The clinical course during hospitalization is detailed in Figure 2. Despite the discontinuation of ibuprofen one week prior, the patient’s renal function continued to worsen after admission, with his serum Cr rising to 1.86 mg/dL on day 15. To establish a definitive diagnosis, a kidney biopsy was performed on the same day under ultrasound guidance, yielding three cores of renal tissue containing 39 glomeruli, with no immediate complications. Given the progressive decline in renal function and the strong clinical suspicion of DI-AIN, the decision was made to initiate empirical treatment. Consequently, oral prednisolone (PSL) at a dose of 40 mg/day (0.8 mg/kg/day) was commenced on the evening of day 15, before the availability of the biopsy results. Following the initiation of steroid therapy, his serum Cr level showed a clear improving trend, decreasing to 1.56 mg/dL by day 23. He was discharged on the same day with his PSL tapered to 30 mg/day.

**Figure 3:**
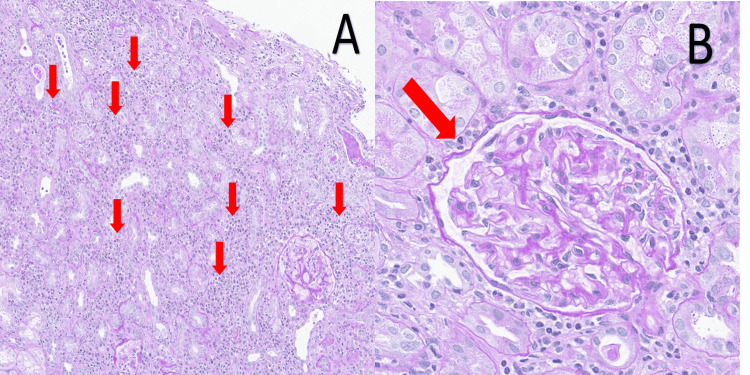
Histopathological findings of the kidney biopsy (periodic Acid-Schiff stain) (A) A medium-power view of the renal cortex demonstrates significant tubulointerstitial changes. There is prominent interstitial infiltration by inflammatory cells (red arrows), accompanied by tubular epithelial cell injury (tubulitis) and expansion of the interstitial space due to edema. These findings are characteristic of acute interstitial nephritis. (B) A high-power view reveals a glomerulus with preserved architecture. The glomerulus appears normocellular, with patent capillary loops (red arrow) and no evidence of mesangial expansion, endocapillary hypercellularity, or other features of glomerulonephritis

Histopathological analysis of the kidney biopsy specimen, completed after the patient's discharge, confirmed the diagnosis. The findings revealed diffuse interstitial infiltration of lymphocytes and eosinophils, associated with tubulitis and edema. The glomeruli were unremarkable. These features were consistent with AIN (Figure 3).

**Figure 4:**
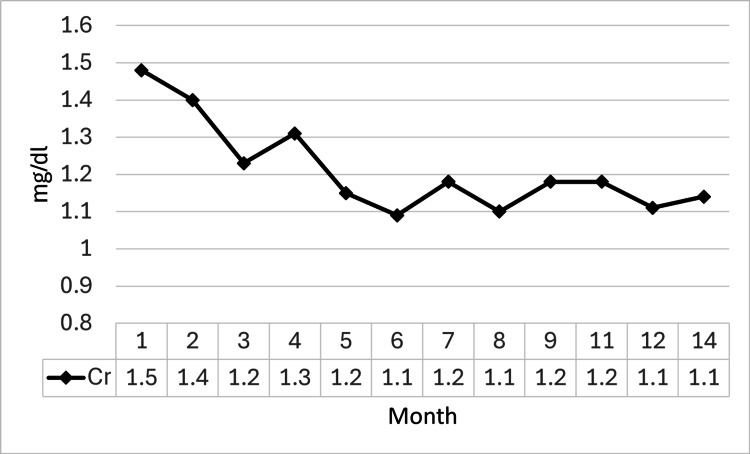
Long-term serum creatinine trend during outpatient follow-up During outpatient follow-up, the serum Cr level continued to improve, gradually decreasing and stabilizing at approximately 1.1 mg/dL from the sixth month onwards during the period of prednisolone tapering Cr: creatinine

The patient’s long-term outpatient course is illustrated in Figure 4. He was managed with a systematic taper of PSL, which was ultimately discontinued approximately 10 months after initial treatment (day 324). His renal function demonstrated sustained recovery; the serum Cr level decreased to 1.09 mg/dL by day 149 and subsequently remained stable at approximately 1.1 mg/dL, with no exacerbation after PSL discontinuation (1.14 mg/dL on day 443).

## Discussion

The mechanisms of NSAID-induced renal injury can be broadly classified into two main pathways. The first is hemodynamic renal injury, which results from the inhibition of intrarenal prostaglandin (PG) synthesis mediated by cyclooxygenase (COX) inhibition [4,5]. The second, more relevant to this case, is allergic DI-AIN. This is primarily considered a delayed-type hypersensitivity response. While the precise pathophysiology is not fully elucidated, it is hypothesized to involve a T-lymphocyte-predominant cellular immune response, leading to cellular infiltration and inflammation in the renal interstitium [6]. The risk of DI-AIN varies depending on the type of NSAID, dosage, duration of administration, and patient-related factors, with the risk reportedly being three to four times higher in patients with CKD [7].

The clinical presentation of DI-AIN is often non-specific, making the diagnosis challenging. While the classic triad of fever, rash, and peripheral eosinophilia is present in less than half of cases [8], many patients, including ours, may present with only systemic symptoms or be asymptomatic. Therefore, a meticulous medication history is crucial when encountering unexplained AKI. In this case, urinalysis on admission was pivotal: despite only mild proteinuria (UPCR 0.24 g/gCr) and a lack of findings suggestive of active glomerulonephritis, markedly elevated levels of multiple urinary tubular injury markers (NAG, β2MG, and L-FABP) provided strong evidence for DI-AIN. Furthermore, the negative autoimmune serology [antinuclear antibody (ANA), antineutrophil cytoplasmic antibody (ANCA)] helped to exclude other causes of interstitial nephritis, thereby strengthening the clinical suspicion of a drug-induced etiology. These markers are particularly useful for identifying tubular damage, especially when glomerular proteinuria is minimal.

To establish a definitive diagnosis, a kidney biopsy was performed on day 15 under ultrasound guidance, yielding three cores of renal tissue containing 39 glomeruli, with no immediate complications. Given that renal function did not improve after ibuprofen discontinuation and DI-AIN was strongly suspected based on the urinalysis findings, empirical steroid therapy was deemed necessary. Therefore, oral PSL at a dose of 40 mg/day (0.8 mg/kg/day) was initiated immediately after the biopsy procedure on day 15, without awaiting the histopathological results. The histopathological findings, which became available post-discharge, subsequently confirmed AIN with marked interstitial infiltration of lymphocytes and eosinophils, tubulitis, and interstitial edema. This retrospectively validated the appropriateness of the early, clinically-guided therapeutic intervention. Even when results are obtained post-treatment, a kidney biopsy remains indispensable for establishing a definitive diagnosis and guiding further management.

The mainstay of DI-AIN treatment is the prompt withdrawal of the offending drug. However, as demonstrated in our patient, whose serum Cr improved after PSL initiation, steroid therapy is a cornerstone when drug withdrawal alone is insufficient [9]. The long-term prognosis of DI-AIN is generally favorable, with a high rate of renal recovery, particularly in drug-related cases [10,11]. While steroid use remains a topic of discussion, evidence suggests it may reduce progression to End-stage kidney disease (ESKD) and improve long-term outcomes in certain subsets [11,12,13]. In our case, the good response to steroids suggested the presence of reversible renal lesions, and early treatment likely contributed to preventing progression to ESKD [14]. The optimal steroid regimen, however, remains to be established and requires individualization [15].

This report also highlights the critical importance of patient education regarding appropriate NSAID use. When common symptoms like fever persist or worsen despite NSAID self-medication, the possibility of drug-induced adverse events should be considered. Prompt consultation with a healthcare professional, rather than continued self-medication, is crucial. Fostering awareness that even over-the-counter NSAIDs can cause serious harm with inappropriate use is a significant public health challenge. Future research should focus on elucidating DI-AIN pathophysiology, validating biomarkers for treatment response, and establishing standard steroid protocols.

The limitations of this report include its single-case nature, precluding the generalization of the therapeutic response. Secondly, other potential contributors to the initial renal dysfunction, such as an undiagnosed viral infection, cannot be entirely excluded. Thirdly, definitive conclusions on the optimal steroid regimen cannot be drawn from this single case. Fourthly, while the initial values of urinary tubular markers were crucial for diagnosis, their utility in monitoring treatment response could not be assessed in this case, as systematic follow-up measurements were not performed. Despite these limitations, this report highlights the diagnostic value of urinalysis with tubular markers; the rationale for early, clinically guided therapeutic intervention; and the importance of a subsequent biopsy-confirmed diagnosis in managing NSAID-associated AIN, thereby offering valuable lessons for clinicians.

## Conclusions

This report underscores the potential for over-the-counter NSAIDs to induce severe AIN. It highlights the diagnostic value of urinary tubular markers in early detection and affirms the indispensable role of renal biopsy for confirmation. Our experience suggests that in patients with high clinical suspicion, prompt initiation of empirical steroid therapy immediately following biopsy, without awaiting histopathological results, is a viable strategy that can lead to significant renal recovery. This underscores the importance of early, clinically-guided intervention in managing NSAID-induced AIN.

## References

[1] Gani D, Parveen P, Saha D (2022). Usage and prescribing patterns of NSAIDs in different general and specialized hospitals in Bangladesh. Sch Acad J Pharm.

[2] Gondane A, Pawar D (2024). Physician perspectives on non-steroidal anti-inflammatory drugs: a comprehensive survey on usage and preferences. Int J Basic Clin Pharmacol.

[3] Burukoglu D, Baycu C, Taplamacioglu F, Sahin E, Bektur E (2016). Effects of nonsteroidal anti-inflammatory meloxicam on stomach, kidney, and liver of rats. Toxicol Ind Health.

[4] Drożdżal S, Lechowicz K, Szostak B (2021). Kidney damage from nonsteroidal anti-inflammatory drugs-myth or truth? Review of selected literature. Pharmacol Res Perspect.

[5] Kikuchi S, Togo K, Ebata N, Fujii K, Yonemoto N, Abraham L, Katsuno T (2021). A retrospective database study of gastrointestinal events and medical costs associated with nonsteroidal anti-inflammatory drugs in Japanese patients of working age with osteoarthritis and chronic low back pain. Pain Med.

[6] Lucas GNC, Leitão ACC, Alencar RL, Xavier RMF, Daher EDF, Silva Junior GBD (2019). Pathophysiological aspects of nephropathy caused by non-steroidal anti-inflammatory drugs. J Bras Nefrol.

[7] Zhang X, Donnan PT, Bell S, Guthrie B (2017). Non-steroidal anti-inflammatory drug induced acute kidney injury in the community dwelling general population and people with chronic kidney disease: systematic review and meta-analysis. BMC Nephrol.

[8] Clive DM, Clive PH (2021). Nonsteroidal antiinflammatory drugs and opioids in chronic kidney disease. Chronic Renal Disease.

[9] Marzec M (2023). Improvement in renal function after empirical steroid therapy in NSAID-induced acute kidney injury. Health Sci Q.

[10] Ramos GA, Ramos MI (2023). NSAID-induced tubulo-interstitial nephritis, case report. South Asian Res J Pharm Sci.

[11] Miao J, Thongprayoon C, Krisanapan P, Buglioni A, Craici IM, Cheungpasitporn W (2024). Clinicopathological characteristics and kidney outcomes in biopsy-confirmed acute interstitial nephritis. Kidney Int Rep.

[12] Prendecki M, Tanna A, Salama AD (2017). Long-term outcome in biopsy-proven acute interstitial nephritis treated with steroids. Clin Kidney J.

[13] Badurdeen Z, Ratnatunga N, Abeysekera T (2023). Randomized control trial of prednisolone and doxycycline in patients with acute interstitial nephritis of unknown aetiology. Trials.

[14] Abuduwupuer Z, Lei Q, Liang S (2023). The spectrum of biopsy-proven kidney diseases, causes, and renal outcomes in acute kidney injury patients. Nephron.

[15] Yaqub S, Aziz A, Awan S (2020). Clinical presentation and outcomes of acute interstitial nephritis: a ten years' experience from a tertiary care hospital in Karachi, Pakistan. Nephrol Dial Transplant.

